# Brain virome dysbiosis in Parkinson’s disease and multiple system atrophy

**DOI:** 10.3389/fmicb.2025.1683277

**Published:** 2025-10-22

**Authors:** Mahin Ghorbani, Giorgio Gabarrini, Zamaneh Hajikhezri

**Affiliations:** 1 Division of Pathology, Department of Laboratory Medicine, Karolinska Institutet, Huddinge, Sweden; 2 Department of Dental Medicine, Karolinska Institute, Stockholm, Sweden; 3 Division of Infectious Diseases, ANA Futura Laboratory, Department of Medicine Huddinge, Karolinska Institutet, Stockholm, Sweden

**Keywords:** brain virome, Parkinson’s disease, multiple system atrophy, brain dysbiosis, putamen

## Abstract

Viral elements have been reported in human brain tissue, yet their presence in the putamen—a region critically affected in Parkinson’s disease (PD) and multiple system atrophy (MSA)has not been characterized. We analyzed whole-genome sequencing data from 32 post-mortem putamen samples (PD: *n* = 10; MSA: *n* = 10; healthy controls: *n* = 12) available under NCBI BioProjects PRJNA756274, PRJNA563007, PRJNA321439, PRJNA555211, and PRJNA555099. Using MetaPhlAn4 for virome profiling, LEfSe for biomarker discovery, and Wilcoxon and ROC analyses for validation, we found that neurodegenerative samples exhibited significantly higher virome alpha diversity compared to healthy controls. LEfSe analysis revealed nine viral species enriched in the neurodegenerative group, including *Pestivirus A, Pestivirus Giraffe-1, Woolly monkey sarcoma virus, Abelson murine leukemia virus, Murine osteosarcoma virus, Human endogenous retrovirus K, Salmonella virus SP6, Taterapox virus*, and *Saccharomyces cerevisiae killer virus M1* (LDA score >2; *p* < 0.05). In contrast, *Alcelaphine gammaherpesvirus 1* was more abundant in controls. While the functional roles of these viruses in the brain remain to be established, several have been previously linked to immunomodulatory effects, suggesting possible relevance to neurodegenerative disease processes. This pilot study provides the first evidence of a brain virome in the human putamen and suggests a potential link between virome dysbiosis and neurodegenerative disease. Distinct viral signatures identified in PD and MSA may serve as candidate biomarkers for early detection and diagnosis.

## Background

Parkinson’s disease (PD) and multiple system atrophy (MSA) are neurodegenerative illnesses defined by an abnormal accumulation of alpha-synuclein aggregates in neurons, nerve fibers, and glial cells (Srinivasan et al., 2021; Reddy and Dieriks, 2022; Woerman et al., 2018). Both conditions are characterized by movement and coordination deficiencies (Mazzoni et al., 2012; Krismer and Wenning, 2017; Wenning et al., 2022; Verschueren et al., 1997). Over years, the global prevalence of Parkinson’s disease has more than doubled, with an estimated 8.5 million people suffering from the disease in 2019. Disability and fatality rates from Parkinson’s disease are rising faster than for any other neurological disorder. Parkinson’s disease, in fact, is expected to have caused 5.8 million disability-adjusted life years in 2019, an 81% rise since 2000 (World Health Organization, 2022). On the other hand, multiple system atrophy is an adult-onset, sporadic, quickly progressing, multisystem, neurodegenerative, deadly disease of unknown cause with different degrees of Parkinsonian symptoms with an incidence of between 1.9 and 4.9 cases per 100,000 persons (Vanacore et al., 2001; Jellinger, 2018).

Despite the identification of numerous genetic, environmental, and microbial contributors, Parkinson’s disease and MSA are increasingly recognized as multifactorial disorders with complex etiologies, in which no single primary cause has been established. In recent years, scientists have proven that some non-brain localized microbiomes such as the salivary microbiome, via the oral-neuro axis, and the gut microbiome, via the gut-brain axis, play a role in the etiology of MSA and PD (Romano et al., 2021; Tan et al., 2017; Jo et al., 2022). However, human and animal model research are insufficient to determine whether the brain has its own microbiome. The term “microbiome” is being used more frequently to describe the complex ecology of microorganisms present naturally within and on a healthy human body. Although the microbiome contains bacterial, viral, and fungal microorganisms, most studies focus on the bacterial component of the microbiome, while the plethora of viruses that inhabit healthy people, known as virome, is significantly less understood, leaving the viral ‘dark matter’ of the oral and gut cavity and their role in neurological disorders largely unexplained.

Investigations revealed a relationship between the virus and alpha-synucleinopathies, including Parkinson’s disease and MSA (Tulisiak et al., 2019). Infections such as influenza, herpes simplex virus, and hepatitis B and C have been associated with an increased risk of Parkinson’s disease (PD), particularly in individuals with severe post-infectious complications such as pneumonia (Cocoros et al., 2021; Lai et al., 2017; Wijarnpreecha et al., 2018; Choi et al., 2020). It is important to emphasize that PD and MSA are multifactorial disorders, and infections by themselves are unlikely to represent causal factors. Robust correlations between viral infection and neurodegeneration have also been reported in other contexts, including enteroviruses in amyotrophic lateral sclerosis (ALS) (Xue et al., 2018; Arru et al., 2021) and herpesviruses such as HSV and HHV-6 in multiple sclerosis (MS) (Khalesi et al., 2023). These examples highlight that viral exposures may act as risk modifiers or immune triggers in complex neurodegenerative pathways, rather than as singular causes. Despite extensive evidence that endogenous retroviruses account for 8% of the human genome and the importance of viral infections in brain diseases (Xue et al., 2020; Li et al., 2015; Grandi and Tramontano, 2018; Phan et al., 2021), research into the presence of a brain virome and its potential role in brain disorders through virome dysbiosis remains limited. However, recent studies have identified viral presence in specific brain regions and suggested an increased risk of neurodegenerative diseases (Ghorbani, 2023; Balakrishnan et al., 2023).

This pilot study hypothesizes the existence of a resident virome in the brain, which refers to the brain virome that has coevolved with humans and persists even when it is not actively replicating, and whose diversity and balanced composition are essential for a healthy brain. This hypothesis presents a distinct contrast to brain abscesses or encephalopathies, as those conditions are unequivocally characterized by the proliferation of well-defined pathogenic microbes. To explore whether latent or endogenous viral elements within the brain may interact with the human genome and whether their balance or dysbiosis could influence brain health and disease, an area that remains largely hypothetical and requires further investigation. This study compares putamen tissue, a brain-specific structure, from healthy controls (HCs) and from patients of neurodegenerative diseases (NDs) such as Parkinson’s disease (PD) and multiple system atrophy (MSA). The choice to use putamen tissue as a sample in this study is based on previous evidence demonstrating significant differences in individuals with Parkinson’s disease (PD) and multiple system atrophy (MSA). For example, individuals with PD exhibited a reduction in the amplitude of low-frequency fluctuations of blood oxygenation-level-dependent signals in the putamen, when compared to healthy controls (Wang et al., 2020).

Moreover, PD was associated with alterations in the putamen’s functional connectivity, including decreased connectivity with the orbitofrontal gyrus and cerebellum and increased connectivity with the supplementary motor area (Shen et al., 2020). D has been associated with alterations in the size of putamen dopamine transporter/synuclein complexes (Longhena et al., 2018). Lastly, evidences demonstrated the existence of early cellular pathways and network alterations in oligodendrocytes in Parkinson’s disease and multiple system atrophy (Azevedo et al., 2022). By comparing the metaviromes of individuals from different groups, this study aims to identify viral members of the microbiome that may contribute to dysbiosis and the development of neurodegenerative diseases. The investigation of virome dysbiosis in PD and MSA could provide insights into the potential involvement of viral factors in these disorders and shed light on the interplay between the virome, host genetics, and metabolic pathways in further studies.

## Materials and methods

### Data collection

In this study, raw data of putamen tissue samples were obtained from the NCBI SRA database^1^ from BioProject: PRJNA756274, PRJNA563007, PRJNA321439, PRJNA555211, PRJNA555099. Supplementary Table 1 contains information about the selected samples, such as NCBI accession number, age, gender, read length (bp), and base pairs and sample quality criteria. For the present analysis, we assembled a total of thirty-two postmortem striatal samples from publicly available BioProjects in the NCBI Sequence Read Archive. The cohort consisted of twelve healthy controls and twenty neurodegenerative disease cases, including ten Parkinson’s disease (PD) and ten multiple system atrophy (MSA) donors. Control material was derived primarily from the bipolar disorder dataset (PRJNA321439) (MacMullen et al., 2017), which provided caudate nucleus and putamen samples from four healthy individuals without major psychiatric or substance-use disorders. Additional control putamen tissue was included from PRJNA563007 (Xicoy et al., 2020), representing pooled samples from non-neurological donors, and from PRJNA756274 (Lim et al., 2021), representing four donors free from alcohol use disorder or psychiatric illness. Together, these resources provided twelve striatal control samples.

The PD dataset was obtained from PRJNA555099, generated at the Harvard Brain Tissue Resource Center, which contains putamen RNA-seq from ten donors (ages 70–87 years) with documented disease duration and neuropathological confirmation of PD. The RNA quality of these samples was high, with RNA integrity numbers (RIN) ranging from 7.3 to 8.7 and postmortem intervals (PMI) between 5 and 27 h. The MSA dataset was drawn from PRJNA555211, contributed by the Bispebjerg Brain Bank, which profiled putamen tissue from ten donors with clinically and pathologically confirmed MSA (ages 56–74 years). These samples also demonstrated good RNA quality, with RIN values ranging from 5.8 to 9.6 (average ≈ 8.1) and PMI between 22 and 72 h.

An additional independent PD dataset was included as a validation cohort from PRJNA845531, (Irmady et al., 2023) which was generated from postmortem striatal tissue provided by the NIH NeuroBioBank. This dataset comprised ten donors with pathologically confirmed PD, including both caudate nucleus and putamen specimens. Donors had an average age of 69 years and an average disease duration of 9 years. RNA integrity was variable, with a mean RIN of approximately 6.0, and PMI ranged from 4.8 to 25.5 h. Clinical metadata indicated that several donors exhibited dementia or dyskinesia, and medication histories were available for both dopaminergic and dementia-related treatments. These samples, having been successfully used for bulk RNA-seq and proteomic profiling in the original study, provide a valuable resource for independent validation of striatal molecular signatures.

By drawing on several independent BioProjects, we established a balanced cohort comprising twelve controls and twenty disease cases spanning PD and MSA. This approach allowed us to evaluate the presence of virome signatures across independent cohorts of neurodegenerative disease. All selected datasets were generated under standardized protocols, had undergone prior peer-reviewed transcriptomic analyses, and consistently reported acceptable RNA integrity and sequencing depth, supporting their suitability for virome-specific RNA analysis.

### Bioinformatics and statistical analysis

FastQC v0.11.8 was used to verify the quality of raw RNA-Seq data. Cutadapt v2.8 was used to eliminate adaptor sequences and low-quality bases from raw data. The preprocessed sequencing data were processed using MetaPhlAn4 (Beghini et al., 2021), which relies on unique clade-specific marker genes discovered from 17,000 reference genomes (13,500 bacterial and archaeal, 3,500 viral, and 110 eukaryotic taxa). To exclude bacterial, eukaryotic (human), and archaeal taxa, the functions “—ignore bacteria,” “—ignore eukaryotes,” and “—ignore archaea” were employed. Taxonomic assignments were made using the internal MetaPhlAn4 database. The feature count table was filtered to eliminate counts >2 with sample prevalence >10%. The final feature count table for downstream analysis was prepared by total-sum scaling (TSS) normalization followed by rarefication for sample depth normalization. Alpha diversity metrics such as Observed, Shannon, Simpson, as well as differential viral communities (beta diversity) between HCs and NDs groups using the Bray-Curtis and Jaccard index distances based on non-metric multidimensional scaling (NMDS) and the PERMANOVA significance test, were calculated in R using the vegan package v2.5.6 (Oksanen et al., 2025). Linear discriminant analysis effect size (LEfSe v1.1.01) (Segata et al., 2011) (LDA score >2, and *p* < 0.05) was used to detect differentially abundant viral species between NDs and HCs groups. The (unpaired)Wilcoxon rank-sum test was used to validate the viral signatures. The receiver operating characteristic analysis (ROC) was used to estimate the predictive value of each discovered viral species. Spearman correlation was used for correlation analysis. Heatmaps of the core virome were created in Microbiome Analyst server (Dhariwal et al., 2017). Finally, to improve the accuracy of biomarker detection, the study used CombiROC, a tool for combining multiple markers, to identify the best combination of viral species for distinguishing between neurodegenerative disease patients and healthy controls. CombiROC uses a machine learning approach to identify the optimal combination of biomarkers that provides the highest sensitivity and specificity (Mazzara et al., 2017).

## Results

### Taxonomic distribution and core virome profiles of NDs and HCs samples

**Figure 1:**
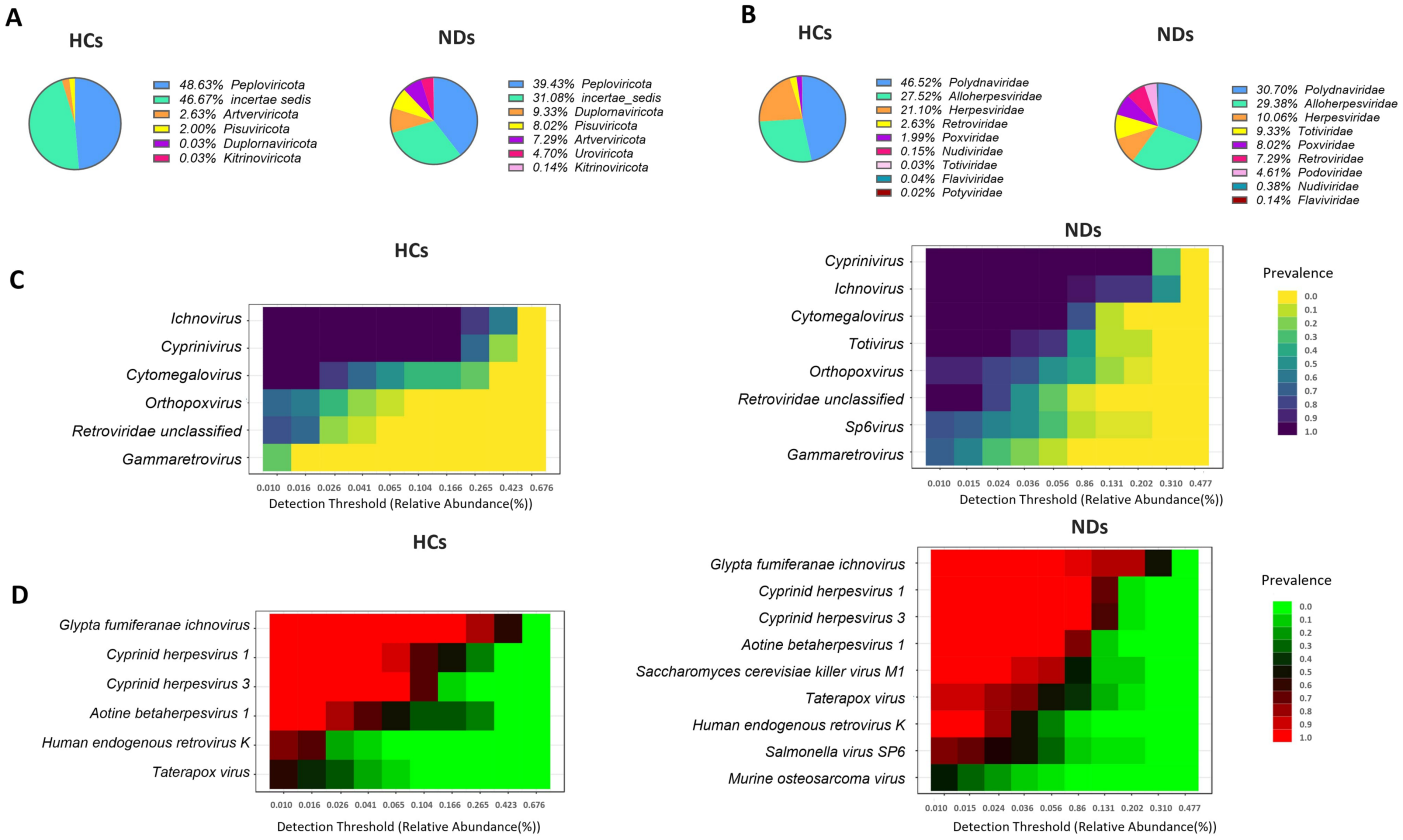
Brain virome composition profiles and core brain virome. **(A)** Pie chart depicting the viral species distribution at the phylum level in HCs and NDs. **(B)** Pie chart illustrating the family-level distribution of viral species in HCs and NDs. **(C)** Heatmap of the core virome of HCs and NDs at the genus level. **(D)** Heatmap of the core virome of HCs and NDs at the species level.

In general, 1,076,756,359 sequence reads were collected from 32 samples, with a range of 5,495,047 to 83,300,086 and an average of 33,648,636 reads. The total number of viruses from all samples was 4,078,840, with an average of 127,463 viruses per sample. Following a 10% prevalence and reads count 2 > filtration, 106 virus types were recovered and assigned to nine phyla, eleven class, eleven order, fourteen families, twenty-seven genera, and thirty four known species. All samples had achieved a plateau, as indicated by the rarefaction curves. All samples have greater than 99.97% Good’s coverage *Peploviricota* and *incertae sedis* were the most common phyla in both groups, (HCs: 49 and 47%, NDs: 39 and 31%, respectively). NDs profiled with 9.33% *Duplornaviricota*, while HCs had only 0.03% of this phylum in their profile. Similarly, NDs profiled with 8% of *Pisuviricota* and 7.29% of *Artverviricota*, while these two phyla were 2 and 2.63% in HCs profile, respectively (Figure 1A). At family level, the interactive pie chart reveals that *Polydnaviridae, Alloherpesviridae* and *Herpesviridae* are the most abundant families in both groups (HCs: 48, 27, 21%; NDs: 31, 29,10%, respectively). Further, NDs profiled with 9.33% of *Totiviridae* and 8% *Poxviridae,* which were only 0.03 and 2% present in the HCs, respectively (Figure 1B). The threshold of the prevalence of genus and species in core virome was set at 20% of samples in each group, with a minimum abundance of 0.01%. NDs’ core virome had eight genera and nine species of taxa, while the HCs ‘s core virome included six genera and six species of taxa. *Ichnovirus, Cyprinivirus*, and *Cytomegalovirus* are the most prevalent in both groups, with 100% prevalence in both groups. NDs had a 100% prevalence of *Totivirus*, which is completely absent in HCs (Figure 1C). According to heatmaps of the dominant core virome at the species level, the most prevalent species are G*lypta fumiferanae ichnovirus, Cyprinid herpesvirus 3, Cyprinid herpesvirus 1, Aotine betaherpesvirus 1,* and *Human endogenous retrovirus K* in both groups. NDs showed 100% prevalence of *Saccharomyces cerevisiae killer virus,* 75% of *Salmonella virus SP6* and 45% of *murine osteosarcoma virus* while, based on 20% prevalence cut off, these species are absent in HCs (Figure 1D).

### Virome richness and diversity in participants’ putamen region

**Figure 2:**
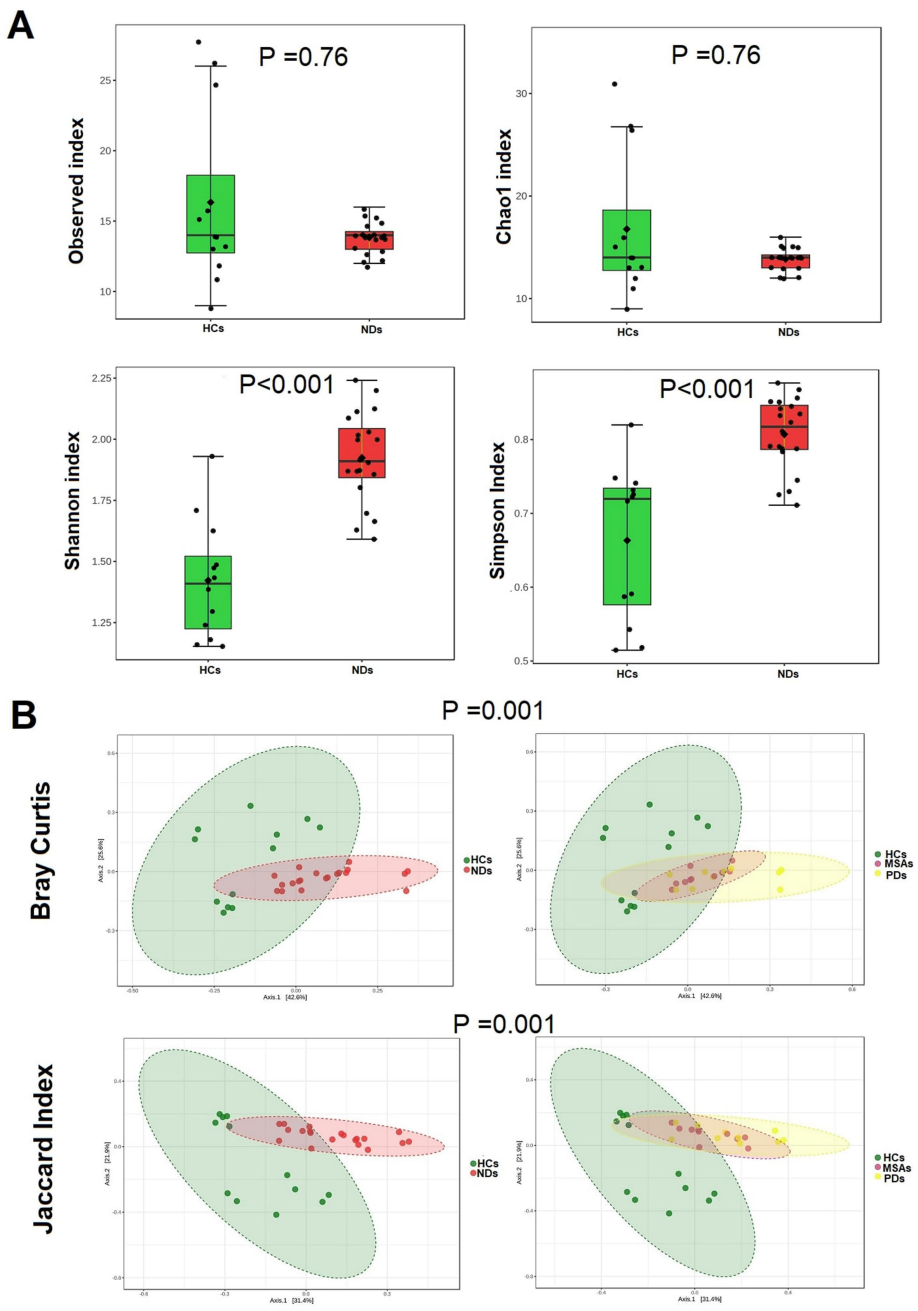
The brain virome richness and diversity and interpersonal variations in HCs and NDs. **(A)** Boxplot of alpha diversity of Observed and Chao1, Shannon and Simpson’s indices reflect the abundance and diversity of OTU in the samples. **(B)** PERMANOVA-validated non-metric multidimensional scaling (NMDS) beta diversity depicted with Bray-Curtis and Jaccard index distances.

The richness of the brain virome varied considerably between HCs and NDs. The NDs demonstrated greater viral diversity than the HCs, (Shannon index *p* < 0.001, and Simpson’s index *p* < 0.001) (Figure 2A). Bray-Curtis and the Jaccard index distances based on non-metric multidimensional scaling (NMDS) revealed interpersonal differences between HCs and NDs (Bray Curtis and PERMANOVA: *p* = 0.001; Jaccard index and PERMANOVA: *p* = 0.001), (Figure 2B).

### Taxonomic differences of brain virome between HCs and NDS

**Figure 3:**
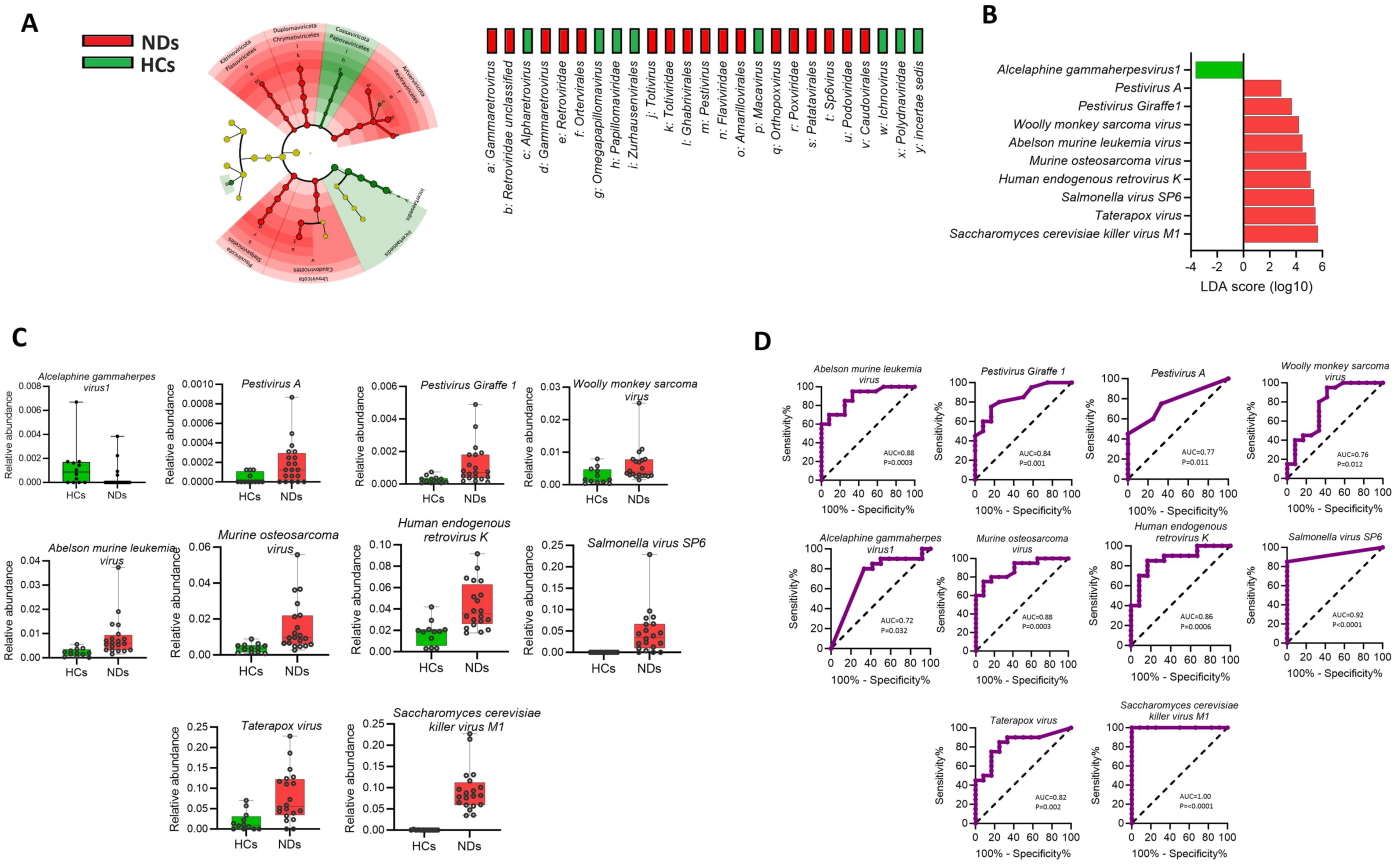
Brain viral signatures are significantly different between HCs and NDs **(A)** Cladogram of the LEfSe analysis of the brain virome, with taxa enriched in HCs in green and those enriched in NDs in red. **(B)** Linear discriminant analysis (LDA) effect size analysis (LEfSe) identified the most differentially abundant viral species between HCs and NDs (*p* < 0.05; LDA score 2). HCs-associated viral species are indicated with negative LDA scores (green) while NDs-associated viral species are indicated with positive LDA scores (red color). **(C)** Mann–Whitney test for each of the identified viral signature displayed (*p* < 0.05). **(D)** Receiver operating characteristics (ROC) analysis showed that the area under curves (AUC) values of the individual viral signature differ between HCs and NDs (AUC; 95% CI).

The Cladogram of Linear discriminant analysis Effect Size (LEfSe) found several taxa differing significantly between HCs and NDs (Figure 3A). LEfSe analysis showed that compared to HCs, NDs had a greater abundance of nine species such as *Pestivirus A, Pestivirus Giraffe-1, Woolly monkey sarcoma virus, Abelson murine leukemia virus, Murine osteosarcoma virus, Human endogenous retrovirus K, Salmonella virus SP6, Taterapox virus* and *Saccharomyces cerevisiae killer virus M1* (*p* < 0.05; LDA score 2) while HCs showed a higher abundance of *Alcelaphine gammaherpesvirus1* compared to NDs. (Figure 3B). The Mann–Whitney test for each of the identified viral signature is displayed in Figure 3C (*p* < 0.05).

The results obtained from the receiver operating characteristic (ROC) analysis (Figure 3D), indicated that the area under curve (AUC) scores of all viral signature were above 0.70 and ranked from lowest to highest as follow*: Alcelaphine gammaherpesvirus 1* (0.7292), *Woolly monkey sarcoma virus* (0.7688), *Pestivirus A* (0.7708), *Taterapox virus*, (0.8292), *Pestivirus Giraffe-1* (0.8479), *Human endogenous retrovirus K* (0.8667), *Murine osteosarcoma virus* (0.8854), *Abelson murine leukemia virus* (0.8896), *Salmonella virus SP6* (0.925), *Saccharomyces cerevisiae killer virus M1* (1.00) (AUC; 95% CI, *p* < 0.05).

**Figure 4:**
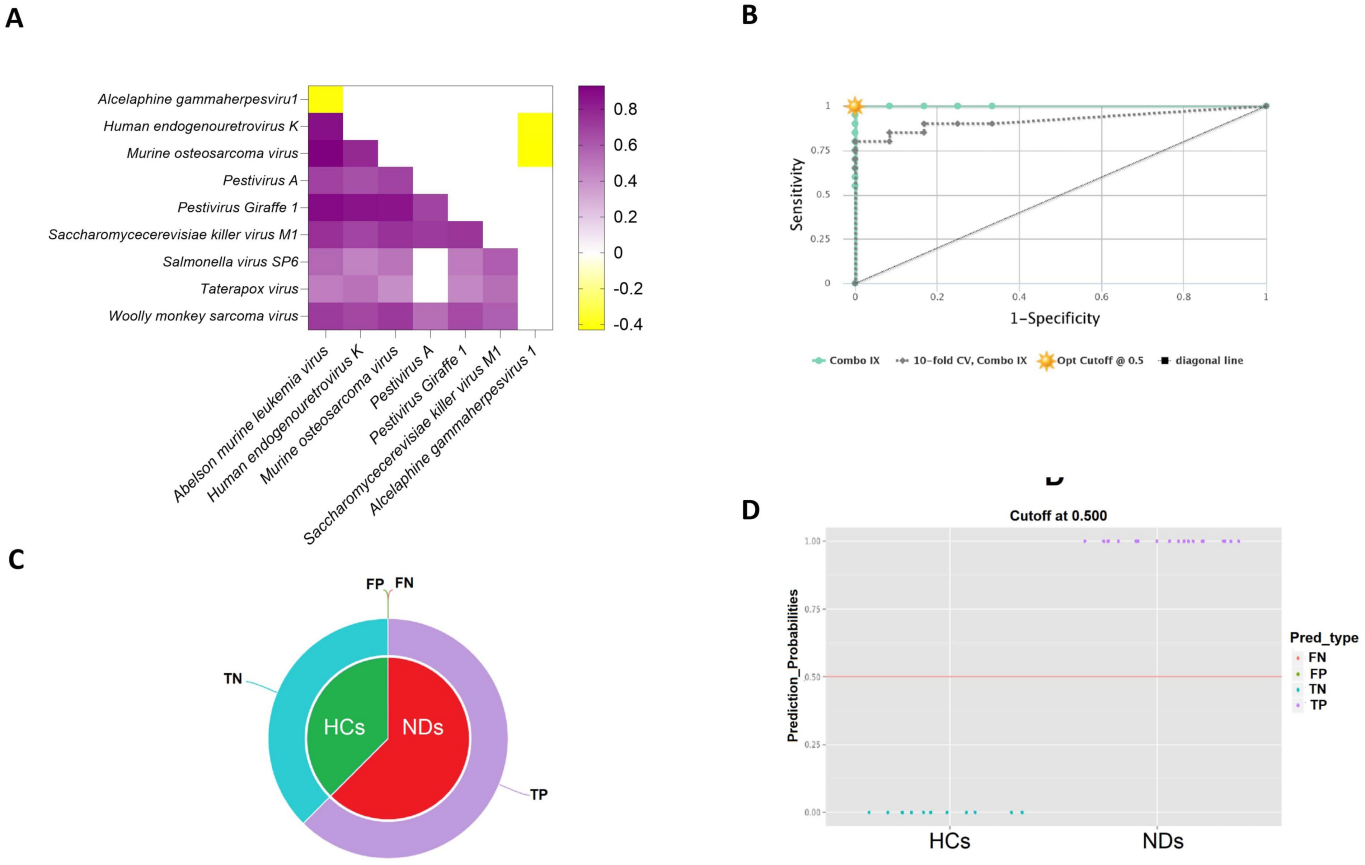
Evaluation of the predictive power of viral signatures identified between healthy controls (HCs) and neurodegenerative disorders (NDs). **(A)** The heatmap represents the co-occurrence of viral signatures identified by Spearman correlation analysis. **(B)** The CombiROC curve combines multiple biomarkers and displays their discriminatory power between the two compared classes. **(C)** The pie charts illustrate the fraction of predictions, including false negatives (FN), false positives (FP), true negatives (TN), and true positives (TP), providing an overall assessment of the predictive performance of the model. **(D)** The violin plot depicts the data’s probability density for HCs and NDs based on the previously determined optimal cut-off on the corresponding ROC curve.

To investigate potential collaborative interactions among the significantly enriched viral species in the pathogenesis of neurodegenerative disorders, Spearman correlation analysis was conducted. The results showed that the viral signatures that were found to be enriched in NDs demonstrated a positive correlation with each other (rho = 0.41 to 0.92, *p* < 0.05). However, these viral signatures displayed a negative correlation with the *Alcelaphine gammaherpesvirus 1* (rho = −0.41 to −0.43, *p* < 0.05), which was enriched in HCs. These findings are presented in Figure 4A.

The CombiROC curve in the study demonstrated an overall value of 1 for the whole cohort and a value of 0.921 for the 10-fold cross-validation, indicating high accuracy in the identification of viral signatures that distinguish HCs from NDs (Figure 4B). The pie chart of CombiROC analysis displayed the absence of false negatives and false positives in the study cohort, which further supports the robustness of the results (Figure 4C). Additionally, the violin plot of CombiROC analysis illustrated the clear separation of HCs and NDs based on the virome data, providing strong evidence for the presence of virome dysbiosis in neurodegenerative disorders (Figure 4D).

**Figure 5:**
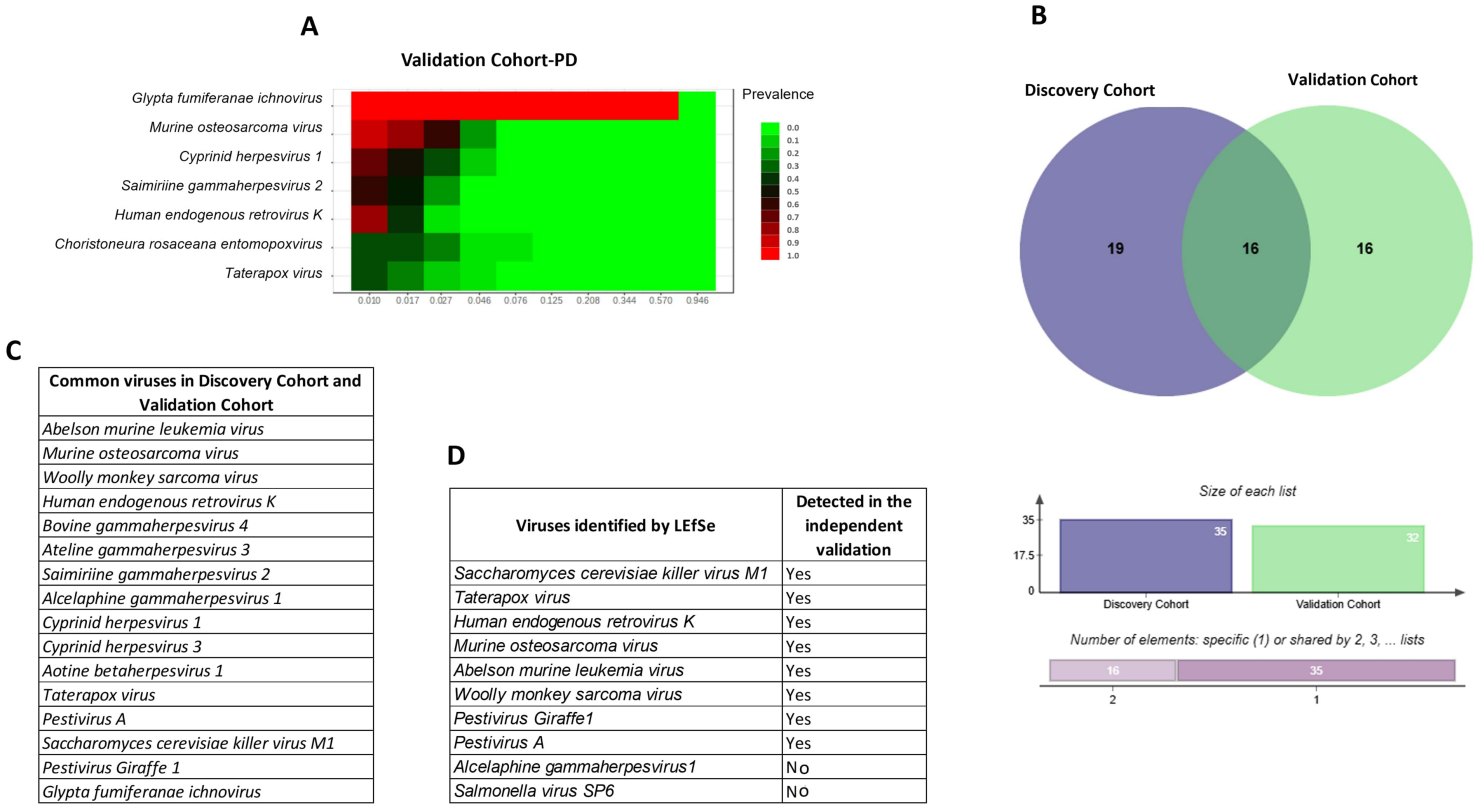
Validation of brain virome signatures in independent cohorts. **(A)** Heatmap showing prevalence of selected viral taxa in the PD validation cohort (PRJNA555099). **(B)** Venn diagram depicting overlap between the discovery cohort (32 striatal samples) and validation cohort (10 PD samples), with 16 viral taxa consistently shared. Bar plots below show the number of unique and overlapping species. **(C)** List of common viral taxa detected in both discovery and validation cohorts, including HERV-K, murine osteosarcoma virus, woolly monkey sarcoma virus, and *Saccharomyces cerevisiae* killer virus M1. **(D)** Viruses identified by LEfSe in the discovery analysis and their detection status in the validation cohort. Together, these analyses demonstrate that a reproducible set of 16 viral taxa is consistently detectable across independent striatal datasets, supporting the existence of a putative core brain virome rather than random or project-specific artifacts.

In the discovery cohort (32 striatal samples), we observed 4,078,840 viral reads (mean 127,463 per sample). In the validation cohort (10 striatal RNA-seq samples), we detected 2,788,750 viral reads (mean 278,875 per sample) and identified 32 viral species. Of these, 16 species were consistently shared across both cohorts (Figures 5A–D). *Abelson murine leukemia virus, Murine osteosarcoma virus, Woolly monkey sarcoma virus, Human endogenous retrovirus K (HERV-K), Bovine gammaherpesvirus 4, Ateline gammaherpesvirus 3, Saimiriine gammaherpesvirus 2, Alcelaphine gammaherpesvirus 1, Cyprinid herpesvirus 1, Cyprinid herpesvirus 3, Aotine betaherpesvirus 1, Taterapox virus, Pestivirus A, Saccharomyces cerevisiae killer virus M1, Pestivirus Giraffe-1*, and *Glypta fumiferanae ichnoviru*s which together accounted for the majority of the viral signal. The recurrence of these taxa across independent striatal cohorts supports the existence of a reproducible core brain virome rather than project-specific contamination.

## Discussion

This pilot study characterized the brain virome in the putamen region of patients with Parkinson’s disease (PD), multiple system atrophy (MSA), and healthy controls (HCs) revealing notable differences in viral composition and diversity. Neurodegenerative disease (ND) samples exhibited significantly greater virome diversity than healthy brains. LEfSe analysis identified nine viral species enriched in NDs, including *Pestivirus A, Pestivirus Giraffe-1, Woolly monkey sarcoma virus, Abelson murine leukemia virus, Murine osteosarcoma virus, Human endogenous retrovirus K (HERV-K), Salmonella virus SP6, Taterapox virus,* and *Saccharomyces cerevisiae killer virus M1*, whereas *Alcelaphine gammaherpesvirus 1* was significantly more abundant in HCs. While prior research has linked viruses such as herpes simplex, hepatitis B and C, and influenza to PD and Alzheimer’s disease Alzheimer’s disease (Cocoros et al., 2021; Wijarnpreecha et al., 2018; Choi et al., 2020; Harris and Harris, 2015), few studies have directly profiled the brain virome in health and disease (Ghorbani, 2023; Balakrishnan et al., 2023). Most microbiome studies have focused on the bacterial components of the oral and gut microbiota. This study is among the first to provide evidence of a potential ‘brain virome dark matter’ and to compare its composition in PD, MSA, and HCs. In this pilot study, the *Herpesviridae* family was more prevalent in healthy controls (HCs) at 21%, compared to 10% in neurodegenerative disease (ND) samples. This observation may align with previous research suggesting an inverse relationship between herpesvirus infections and Parkinson’s disease (PD) risk. Notably, a large U. S.-based population case–control study conducted in 2009 among Medicare recipients aged 66 to 90 (comprising 89,790 PD cases and 118,095 matched controls) found that PD risk was inversely associated with herpes simplex virus infection (OR 0.79; 95% CI 0.74–0.84), *herpes zoster* (OR 0.88; 95% CI 0.85–0.91), and use of anti-herpetic medications (OR 0.87; 95% CI 0.80–0.96). However, the study’s authors emphasized the need for further research to clarify whether this relationship is causal (Camacho-Soto et al., 2020). This study suggests higher abundance of *Herpesviridae* in healthy controls may reflect stable latent infections that help modulate immune activity and potentially reduce neuroinflammatory processes involved in neurodegeneration. This study found a higher prevalence of *Pestivirus* species, particularly *Pestivirus A* and *Pestivirus Giraffe-1*, in neurodegenerative disease (ND) samples. *Pestivirus,* a genus within the *Flaviviridae* family, primarily infects mammals such as cattle, sheep, goats (Bovidae), and swine (Suidae). These viruses are best known for causing hemorrhagic syndromes, reproductive disorders, and fatal mucosal disease in livestock (Oksanen et al., 2025; Segata et al., 2011; Dhariwal et al., 2017; Mazzara et al., 2017; Harris and Harris, 2015; Camacho-Soto et al., 2020; Thür et al., 1998; Beghini et al., 2021; Golender et al., 2021). However, their potential involvement in human neurodegenerative disease remains unexplored. Notably, studies in neonatal animals have shown that *Pestivirus* infection can lead to cerebellar hypoplasia, a condition marked by impaired motor coordination (Thür et al., 1998; Simmonds et al., 2017; Luzzago and Decaro, 2021) suggesting a possible neurotropic or neurodevelopmental impact worthy of further investigation.

In this pilot study, neurodegenerative disease (ND) samples showed a high abundance of retroviral species, including *Woolly monkey sarcoma virus, Abelson murine leukemia virus,* and *Human endogenous retrovirus K* (HERV-K). While direct links between retroviruses and PD or MSA have been limited, accumulating evidence supports a potential role for HERV-K in neurodegenerative diseases (Adler et al., 2024; Gröger et al., 2021). While it is unlikely that viruses adapted to non-human hosts (e.g., pestiviruses in ruminants, yeast viruses, or bacteriophages) directly infect human neurons, their detection in putamen tissue may still be biologically relevant. First, endogenous retroviral elements of non-human ancestry (e.g., HERV-K) remain transcriptionally active in neurodegeneration and modulate immune responses (Xue et al., 2020). Second, non-replicating viral proteins, such as those from yeast or phages, can act as molecular mimics, shaping host immunity or triggering inflammatory cascades (Mihalič et al., 2023). Third, zoonotic viruses illustrate how non-human pathogens can occasionally cross species boundaries and leave molecular traces in human tissue (Jiang et al., 2023). Taken together, these considerations suggest that such viral sequence signatures may represent latent genomic fragments, immune-modulatory bystanders, or cross-species viral ancestry rather than active infection. Nevertheless, their consistent detection across multiple BioProjects supports that they are unlikely to be random artifacts. Future orthogonal validation (*in situ* hybridization, immunohistochemistry, qPCR, electron microscopy) will be required to establish whether these viral signals are truly present in brain tissue and whether they influence disease-associated immune tone.

HERV-K RNAs and proteins have been shown to be neurotoxic, and its involvement has been implicated in other movement-related disorders, including multiple sclerosis (MS) and amyotrophic lateral sclerosis (ALS). ALS patients, who often present with Parkinsonian features such as tremor, rigidity, and bradykinesia, exhibit elevated levels of HERV-K DNA and RNA. Furthermore, studies have detected increased antibodies against HERV-K in the serum and cerebrospinal fluid (CSF) of ALS patients (Steiner et al., 2022), HERV-K is also capable of modulating the immune system by inducing mediators involved in proinflammatory responses, suggesting a possible immunopathological mechanism in neurodegeneration. Given the role of HERV-K in the pathogenesis of currently incurable diseases, it may be possible to develop novel therapies based on its suppression. In both the discovery and validation cohorts, we consistently detected sequences corresponding to the *Saccharomyces cerevisiae killer virus M1*. While this virus is adapted to yeast hosts and is unlikely to replicate in human neurons, its reproducible detection across independent striatal datasets suggests that it is not a random artifact. Instead, we interpret this finding as a putative viral sequence signature that may represent latent fragments, environmental carry-over, or indirect immune-modulatory elements rather than active infection. Yeast-derived nucleic acids and proteins are known to interact with innate immune pathways (Claudepierre et al., 2014), and it is therefore plausible that such elements could influence host immunity even without replication in brain tissue. Nevertheless, we emphasize that this result should be regarded as hypothesis-generating, and orthogonal validation will be required to determine whether the yeast virus signal reflects a true biological presence or a technical.

An evolutionary perspective may also help contextualize the role of endogenous retroviruses in both autoimmunity and neurodegeneration. For example, HERVs have been proposed as immune modulators in chronic inflammatory diseases such as rheumatoid arthritis (De Francesco, 2024) and recent work suggests that these same retroviral elements may contribute to immune dysregulation and neural dysfunction in neurodegenerative disorders (Censi et al., 2024)This dual role underscores the possibility that HERV activity links systemic autoimmunity and neurodegeneration through shared pathways of inflammation and immune escape.

In our study, G*ammaretroviruses,* including *Murine leukemia virus, Woolly monkey sarcoma virus*, and *Murine osteosarcoma virus*, have been detected in the brains of NDs It has been demonstrated that *Gammaretrovirus* has leukemia-inducing properties and can cause immune system dysfunction, which contributes to Parkinson’s disease (Hunter et al., 2017). Another study showed the increased risks of leukemia among Parkinson individuals with leucine-rich repeat kinase 2 gene (Agalliu et al., 2019). The function of *Gammaretrovirus* in immunosuppression, which may play a role in neurodegenerative disease, however, warrants further study. *Taterapox virus* from the *Orthopoxvirus* genus was found in abundance within the NDs. Neuropsychiatric and neurodegenerative diseases may be influenced by interactions between microbiota and antipsychotic-brain disorder medications, according to research on the gastrointestinal and oral microbiomes (Seeman, 2021; Chen et al., 2020), and it has been demonstrated, in fact, that diazepam exacerbates orthopoxviruses infections and suppresses the immune system (Huemer et al., 2010). The interaction between neurodegenerative treatment medications and brain virome merits additional study.

Beyond the putamen region, other studies have demonstrated correlations between viral signatures and neurodegenerative disease in distinct brain regions. For instance, HHV-6 and HHV-7 were enriched in cortical and hippocampal regions of Alzheimer’s disease brains (Piotrowski et al., 2023; Skuja et al., 2021). Herpes Simplex Virus Type 1 On temporal cortices, frontal cortices, and hippocampus in Sporadic Alzheimer’s Disease (Harris and Harris, 2015),individuals with multiple sclerosis (MS) test positive for EBV, and its presence is particularly noted in MS brain lesions (Orr and Steinman, 2025), Our recent study also investigated the human brain virome in Brodmann Area 46, and showed distinct virome differences between individuals with schizophrenia and healthy controls (Ghorbani, 2023).

These studies provide converging evidence that viral activity across multiple brain regions may contribute to diverse neurodegenerative processes, supporting the concept that virome dysbiosis is not restricted to one anatomical site.

Most viral taxa detected in our dataset were annotated as non-human. While contamination during post-mortem handling or sequencing cannot be excluded, it is important to note that non-human viruses are not necessarily irrelevant to human health. Non-human primates (NHPs) share 75–98.5% genetic homology with humans and exhibit highly similar physiology, tissue structure, immunity, and metabolism, enabling them to serve as natural hosts for many of the same pathogens. This close relationship makes NHP viruses valuable models for studying cross-species transmission, immune modulation, and for developing vaccine (Jiang et al., 2023; Estes et al., 2018). Non-human viruses can significantly impact human health, often through zoonotic spillover or latent persistence (Jiang et al., 2023). For instance, monkey pox virus causes rash illness in humans and is diagnosed by RT-PCR or NGS, yet no curative therapy exists (Khattak et al., 2022). Monkey B virus can induce fatal encephalitis in humans following NHP exposure, with diagnosis relying on PCR and serology; while antivirals can suppress infection, they are not curative (Jiang et al., 2023; Gong et al., 2022). Similarly, Ebola and Marburg viruses cause severe hemorrhagic fevers diagnosed by clinical and serological assays, and although monoclonal antibody therapies such as Inmazeb and Ebanga provide partial protection, no definitive cure exists (Jiang et al., 2023; Cross et al., 2023).

Additional NHP viruses provide valuable insights into human disease. Simian cytomegalovirus (SCMV), a beta-herpesvirus restricted to rhesus macaques, shares close homology with human cytomegalovirus (HCMV). Because SCMV infection produces pathological outcomes resembling HCMV, it has been used as a reliable model for studying viral pathogenesis, immune suppression, and congenital infection. Importantly, efficient natural transmission within macaque colonies has also been leveraged to test vaccine efficacy, underscoring its value in translational research (Jiang et al., 2023; Estes et al., 2018; Yee et al., 2023; Marshall et al., 2019). Other NHP viruses—including simian immunodeficiency virus (SIV), simian hemorrhagic fever virus (SHFV), adenoviruses, and parvoviruses—share close homology with human pathogens and serve as critical models for infection, immunity, and vaccine development (Rollier et al., 2011; Chavda et al., 2023). SIV, for example, closely parallels HIV; adenoviruses are widely used as vaccine vectors; and SHFV models hemorrhagic fever. As highlighted by Jiang et al. (2023), such viruses demonstrate that non-human elements of the virome are not incidental but can inform mechanisms of human disease and therapeutic strategies (Jiang et al., 2023; Gallinaro et al., 2025).

Endogenous retroviruses such as HERV-K further illustrate how viral elements of non-human ancestry can be transcriptionally active in neurodegeneration, directly modulating immune responses and neuropathology (Xue et al., 2020; Li et al., 2015). At the same time, not all viral elements are overtly pathogenic. Human pegivirus (HPgV), for example, is generally regarded as harmless; however, its capacity to modulate immune pathways—and to interact with host genetics such as LRRK2—suggests it could still influence neuroinflammation, mitophagy, and disease progression in Parkinson’s disease (Hanson et al., 2025). Taken together, these findings indicate that non-human, endogenous, and seemingly benign viral elements should not be dismissed as incidental; rather, they may constitute a latent or hidden virome layer capable of shaping immune tone and modulating disease vulnerability. Taken together, these findings indicate that non-human, endogenous, and seemingly benign viral elements should not be dismissed as incidental; rather, they may constitute a latent or hidden virome layer capable of shaping immune tone and modulating disease vulnerability. Nevertheless, we emphasize that contamination during sample handling or sequencing cannot be completely excluded, and therefore these results must be regarded as hypothesis-generating. Future studies employing orthogonal validation approaches such as *in situ* hybridization, immunohistochemistry, qPCR, or electron microscopy will be essential to confirm whether the viral sequences identified here represent true presence in brain tissue and to clarify their potential mechanistic role in neurodegeneration.

It is important to note that not all viral elements detected in the brain necessarily promote pathology. For example, human endogenous retroviruses (HERVs) have been detected at appreciable levels in healthy control brains. HERV-K expression is predominantly localized to astrocytes, where it colocalizes with glial fibrillary acidic protein (GFAP), an intermediate filament protein widely used as a marker of astrocyte activation and injury. Compared to healthy controls, brain samples from PD patients show reduced expression of both HERV-K and GFAP, with similar decreases also observed in peripheral blood. Lower HERV-K expression correlates with greater disease severity, suggesting that loss of astrocytic activity linked to HERV-K may contribute to PD progression (Simula et al., 2025).

Endogenous retroviruses (HERVs) are relics of ancient retroviral infections now fixed in the human genome, beyond being passive remnants, HERVs can act as regulatory elements, contribute to embryogenesis, and in some cases have been co-opted for physiological functions such as placental development (Balestrieri et al., 2019)However, their abnormal reactivation in response to environmental or immune stimuli has been implicated in cancer, autoimmune disorders, and neuropsychiatric and neurodevelopmental conditions, including autism, ADHD, and schizophrenia (Bo et al., 2024; Balestrieri et al., 2014; Soleimani et al., 2024; Yang et al., 2024). These findings underscore that HERV activity can serve both beneficial and detrimental roles, providing a molecular link between genetic susceptibility, environmental triggers, and altered brain development.

some gammaherpesviruses are well-established pathogens in non-human primate, other members of this family have been reported in the brains of healthy individuals without overt disease (Ghorbani, 2023).

This raises the possibility that certain herpesviruses may play non-pathogenic or even protective roles, for example by modulating immune tone. Acknowledging these dual aspects underscores that the brain virome may represent a dynamic ecosystem with both harmful and beneficial viral influences.

The absence of classical neurotropic viruses such as HSV or JC virus in our dataset does not necessarily indicate their absence from the brain. These viruses are well known for their latent or silent infections, often persisting below the detection threshold of untargeted sequencing (Kennedy et al., 2015; Marzocchetti et al., 2005), In addition, neurotropic viruses may show regional specificity, being enriched in other brain areas not analysed here for example Progressive multifocal leukoencephalopathy (PML) is a demyelinating disease caused by JC virus, which preferentially infects oligodendrocytes in the cerebral white matter, especially the parietal, occipital, and frontal lobes, leading to multifocal lesions (Cortese et al., 2021). Our observation that most detected viral sequences are non-human or endogenous is consistent with prior metagenomic surveys, which also report a predominance of latent or ‘hidden’ virome elements rather than overt infections (Ghorbani, 2023; Balakrishnan et al., 2023).

Contrasting results have been reported, including enrichment of HHV-6 and HIV in Alzheimer’s brains and detection of HSV-1 and HHV in multiple sclerosis (Piotrowski et al., 2023; Carneiro et al., 2022; Feng et al., 2025). HSV-1 DNA has also been found in both diseased and non-diseased brains, with studies suggesting impaired HSV-1–specific immunity in Alzheimer’s patients (Rizzo, 2020). Such discrepancies likely reflect differences in brain region, disease stage, or methodological sensitivity.

This pilot study offers novel insights into the human brain virome in the context of neurodegenerative diseases, but several important areas remain to be addressed in future research. While the use of SRA metagenomic data enabled access to brain samples otherwise difficult to obtain, the lack of accompanying clinical metadata (e.g., age, sex, disease stage, treatment history) and the modest sample size limit the ability to control for individual variability. Expanding future analyses to include larger, clinically annotated datasets will strengthen the validity and interpretability of virome associations.

Investigating the brain virome remains inherently challenging, given the restricted access to human brain tissue, which is primarily available post-mortem. Nonetheless, this limitation can be addressed by leveraging well-established animal models of neurodegeneration to explore region-specific virome dynamics under controlled conditions.

It is also important to consider potential technical factors affecting virome detection, including the need for optimized nucleic acid stabilization protocols and the selection of comprehensive viral reference databases. Cross-validating findings using multiple bioinformatics pipelines will enhance the robustness of viral identification.

A key limitation of this study is the relatively small sample size (10 PD, 10 MSA, and 12 controls), which increases the risk of type I error and overfitting. This limitation is especially relevant to the classification analysis (Figure 4), where the apparent perfect separation of groups almost certainly reflects model optimism in a small dataset rather than a generalizable predictive effect. Accordingly, we interpret these results as exploratory in-sample separability only and emphasize that validation in larger, independent cohorts will be required to determine whether any viral signatures have true predictive value.

A further limitation relates to possible microbial contamination during surgery or autopsy, which cannot be fully excluded in publicly available datasets. However, several steps mitigate this concern. First, sequencing was performed under standardized protocols across multiple NCBI BioProjects. Second, our pipeline excluded bacterial, archaeal, and eukaryotic reads and applied strict prevalence and abundance thresholds to reduce low-level contaminants. Third, key viral taxa were reproducibly enriched across independent datasets, making random contamination less likely. Nonetheless, the possibility of contamination highlights the need for future validation studies using prospectively collected, contamination-controlled brain tissue.

Overall, this study sets the stage for a new line of inquiry into the brain virome and its role in neurodegenerative disease. Continued efforts to overcome current challenges will help clarify whether virome composition contributes to disease mechanisms, and may ultimately support the development of new diagnostic tools or targeted antiviral strategies.

## Conclusion

This pilot study supports the hypothesis that a resident brain virome exists and may contribute to the pathogenesis of neurodegenerative diseases. Observed virome dysbiosis in Parkinson’s disease and multiple system atrophy suggests a potential role for viral imbalance in disease onset or progression. These findings highlight a previously underexplored aspect of brain biology and open new avenues for investigating viral–host interactions in neurodegeneration. Further research is essential to confirm these associations, clarify underlying mechanisms, and evaluate the diagnostic or therapeutic potential of brain virome signatures.

## Data Availability

The original contributions presented in the study are included in the article/Supplementary material, further inquiries can be directed to the corresponding author.

## Footnotes

^1^ https://www.ncbi.nlm.nih.gov/sra

## Glossary

ALS: amyotrophic lateral sclerosis
ASCA: anti-*Saccharomyces cerevisiae* antibodies
bp: base pairs
CI: confidence interval
CSF: cerebrospinal fluid
HCs: healthy controls
HERV-K: human endogenous retrovirus K
IgA: immunoglobulin A
IgG: immunoglobulin G
LDA: linear discriminant analysis
LEfSe: linear discriminant analysis Effect Size
MS: multiple sclerosis
MSA: multiple system atrophy
NCBI: National Center for Biotechnology Information
NDs: neurodegenerative diseases
NMDS: non-metric multidimensional scaling
PD: Parkinson’s disease
PERMANOVA: permutational multivariate analysis of variance.
RA: rheumatoid arthritis
ROC: receiver operating characteristic
ScV-M1: *Saccharomyces cerevisiae* killer virus M1
SRA: sequence read archive
TSS: total-sum scaling
WHO: World health organization
