# Correction: Brain virome dysbiosis in Parkinson's disease and multiple system atrophy

**DOI:** 10.3389/fmicb.2025.1731713

**Published:** 2025-11-06

**Authors:** Frontiers Production Office

**Affiliations:** Frontiers Media SA, Lausanne, Switzerland

**Keywords:** brain virome, Parkinson's disease, multiple system atrophy, brain dysbiosis, putamen

The citations MacMullen et al., 2017, Irmady et al., 2023, and Beghini et al., 2021 were erroneously included at certain points in the section **Methods, Bioinformatics and statistical analysis**.

This section now reads as:

“FastQC v0.11.8 was used to verify the quality of raw RNA-Seq data. Cutadapt v2.8 was used to eliminate adaptor sequences and low-quality bases from raw data. The pre-processed sequencing data were processed using MetaPhlAn4 (Beghini et al., 2021), which relies on unique clade-specific marker genes discovered from 17,000 reference genomes (13,500 bacterial and archaeal, 3,500 viral, and 110 eukaryotic taxa). To exclude bacterial, eukaryotic (human), and archaeal taxa, the functions “—ignore bacteria,” “—ignore eukaryotes,” and “—ignore archaea” were employed. Taxonomic assignments were made using the internal MetaPhlAn4 database. The feature count table was filtered to eliminate counts >2 with sample prevalence >10%. The final feature count table for downstream analysis was prepared by total-sum scaling (TSS) normalization followed by rarefication for sample depth normalization. Alpha diversity metrics such as Observed, Shannon, Simpson, as well as differential viral communities (beta diversity) between HCs and NDs groups using the Bray-Curtis and Jaccard index distances based on non-metric multidimensional scaling (NMDS) and the PERMANOVA significance test, were calculated in R using the vegan package v2.5.6 (Oksanen et al., 2025). Linear discriminant analysis effect size (LEfSe v1.1.01) (Segata et al., 2011) (LDA score >2, and *p* < 0.05) was used to detect differentially abundant viral species between NDs and HCs groups. The (unpaired) Wilcoxon rank-sum test was used to validate the viral signatures. The receiver operating characteristic analysis (ROC) was used to estimate the predictive value of each discovered viral species. Spearman correlation was used for correlation analysis. Heatmaps of the core virome were created in Microbiome Analyst server (Dhariwal et al., 2017). Finally, to improve the accuracy of biomarker detection, the study used CombiROC, a tool for combining multiple markers, to identify the best combination of viral species for distinguishing between neurodegenerative disease patients and healthy controls. CombiROC uses a machine learning approach to identify the optimal combination of biomarkers that provides the highest sensitivity and specificity (Mazzara et al., 2017).”

The original version of this article has been updated.

